# Prognostic Value of Immunoglobulin G (IgG) Patterns by Western Blotting Immunodetection in Treated Dogs Previously Infected with *Leishmania infantum*

**DOI:** 10.3390/vetsci8120293

**Published:** 2021-11-27

**Authors:** Ehab Kotb Elmahallawy, Stefania Zanet, Marco Poggi, Khalaf F. Alsharif, Maha S. Lokman, Anna Trisciuoglio, Ezio Ferroglio

**Affiliations:** 1 Department of Zoonoses, Faculty of Veterinary Medicine, Sohag University, Sohag 82524, Egypt; 2 Department of Veterinary Sciences, University of Turin, Via Leonardo da Vinci 44, Grugliasco, 10095 Torino, Italy; stefania.zanet@unito.it (S.Z.); marco.poggi@centroveterinarioimperiese.com (M.P.); anna.trisciuoglio@unito.it (A.T.); 3 Department of Clinical Laboratory Sciences, College of Applied Medical Sciences, Taif University, P.O. Box 11099, Taif 21944, Saudi Arabia; alsharif@tu.edu.sa; 4 Biology Department, College of Science and Humanities, Prince Sattam bin Abdul Aziz University, Alkharj 11942, Saudi Arabia; ms.hussein@psau.edu.sa; 5 Department of Zoology and Entomology, Faculty of Science, Helwan University, Cairo 11795, Egypt

**Keywords:** IgG patterns, Western blotting, canine leishmaniasis

## Abstract

Leishmaniasis is a heterogeneous group of neglected tropical diseases with various clinical syndromes, which is caused by obligate intracellular protozoa of the genus *Leishmania* and transmitted by the bite of a female phlebotomine sandfly. Humans and several animal species are considered as reservoirs of the disease. Among other animal species, dogs are the most important reservoirs in a domestic environment, maintaining the endemic focus of the parasite. The behavior of the disease progression and the clinical symptoms of the disease in the infected dog is mainly associated with depressed cellular immunity and strong humoral response. This study aimed to assess the role of Western blotting in the analysis of the idiotype expression of the two main immunoglobulins (IgG1 and IgG2) in dogs that are naturally infected with *Leishmania infantum* (*L. infantum*) and treated with N-methyl meglumine antimoniate. Interestingly, for the first time, our study identified several *L. infantum* antigen polypeptides (14, 31, 33, 49, 64, 66, 99, and 169 kDa) that more frequently stimulate an immune reaction in recovered dogs after treatment, whereas in the non-recovered group of dogs, four antigen polypeptides of *L. infantum* with molecular weights of 31, 49, 66, and 115 kDa with unfavorable prognosis were identified. Clearly, these interesting findings confirm the strong association between the detected immunodominant bands and the successful recovery in treated dogs that can be used for differentiating the treated dogs from the untreated dogs, as well as the markers of a favorable or unfavorable prognosis and, as a consequence, the prediction of the clinical outcome of the disease. Likewise, these data could be helpful in the implementation of novel vaccines from the detected antigens.

## 1. Introduction

Leishmaniasis is a group of neglected diseases with a clear endemicity in tropical and subtropical areas, caused by the infection by flagellates’ parasites of the genus *Leishmania,* which occur in all inhabited continents except Australia and Antarctica [1,2]. More than 350 million people in 98 countries around the world are at risk of these diseases, with an annual mortality rate higher than 60,000 people [3]. There are three forms of human leishmaniasis: cutaneous leishmaniasis (CL), mucocutaneous leishmaniasis, and fatal visceral leishmaniasis (VL) in the absence of proper treatment [3,4]. The public health and zoonotic importance of leishmaniasis is growing [3,5,6,7]. In this regard, there is a marked increase in the incidence of human infections (co-infection), especially in immunocompromised cases, as in VL/HIV co-infections cases [5,6,7], and new foci have been reported out of the traditionally endemic areas [6,8]. In accordance with its zoonotic concern, leishmaniasis has two zoonotic forms in Europe: zoonotic CL and zoonotic VL (ZVL) [9]. Importantly, ZVL, caused by *L. infantum*, is an endemic form of the disease in all countries of the Mediterranean basin, where VL cases in the area account for approximately 5–6% of the global burden [5,9,10,11]. Among others, *Phlebotomus perniciosus* (sandfly) acts as the main vector, and dogs are the main reservoirs of infection in this area [6,10,12]. Leishmaniasis in canines is a widespread zoonotic disease that is commonly known as canine leishmaniasis, where the seroprevalence may exceed 40% in certain endemic areas [6,13]. Besides being a common life-threatening zoonotic disease, leishmaniasis in dogs is a very complex pathology that is of particular interest to veterinary practitioners [14].

The progression of the disease in infected dogs differs depending on the response of their immune systems [15], and the progression from infection to clinical disease is mainly associated with depressed cellular immunity and ineffective humoral response [15,16,17]. While this condition subsists, dogs with progressive disease may develop lymphadenopathies (93%), dermatitis (90%), onychogryphosis (75%), weight loss (26%), cachexia (24%), locomotion problems (23%), conjunctivitis (18%), and epistaxis (9%) [15,18]. Despite the recent improvements in the diagnosis and the treatment of the disease, they are neither easy nor considerably successful, and can be very distressing for the animals and frustrating for their owners [4,15,19,20,21,22].

Hence, a more specific diagnostic tool and an efficient therapy are still needed. Western blotting (WB) analysis is one of the most sensitive and specific techniques that can improve the diagnostic capabilities for canine and human leishmaniasis [23,24]. On the other hand, IgG1 and IgG2 are the two main immunoglobulins that have been related to host Th1/Th2 responses and disease evolution [15,23,25,26,27,28,29,30,31]. Particularly, IgG1 has been associated with symptomatic infection, whereas IgG2 is more frequently found in asymptomatic dogs [23,26,27,29,30,32]. The present study used WB to analyze the idiotype expression of the total IgG, IgG1, and IgG2 in dogs naturally infected with *L. infantum* and treated with N-methyl meglumine antimoniate, with the aim of detecting the specific immunoglobulins that can be markers of early infection or early symptomatic phases, as well as prognostic markers. Eventually, the specific antigenic fractions could be used to implement a recombinant antigen used to stimulate an effective antibody response in infected dogs.

## 2. Material and Methods

### 2.1. Ethical Considerations

The study was conducted according to the guidelines of the Declaration of Helsinki and approved by the Institutional Review Board of the Department of Veterinary Sciences (local ethical approval), the University of Turin, Italy, under ethical approval number 2021/2.

### 2.2. Study Population

A total of 70 dogs naturally infected with *L. infantum* were included in this study. The dogs, which were of different ages, breeds, and genders, were recruited in Liguria, a highly endemic region of northwestern Italy. The dogs were routinely sampled during veterinary clinic visits and diagnosed with *Leishmania* by veterinary practitioners on the basis of clinical signs, serological tests (IFA), and molecular tests (PCR). The animals that, following ESCCAP (European Scientific Counsel Companion Animal Parasites) guidelines, met the treatment requirements were treated with N-methyl meglumine antimoniate following the standard protocols. All dogs were periodically checked (following the time schedule suggested by ESCCAP guidelines) for a period of 30 months. Successful therapeutic response was achieved upon the complete remission of the clinical signs and negative IFA results for a period of at least 12 months after antimonial therapy. Dogs for whom the complete remission of clinical signs and hematological parameters was not obtained were considered to have an unfavorable prognosis. Whole blood was collected from each animal through the jugular vein puncture in a clean, sterile tube. The samples were then transported to the Laboratory of Parasitology, Department of Veterinary Sciences, the University of Turin, Italy, where they were centrifuged, and the sera isolated and stored at −80 °C until testing.

### 2.3. Clinical and Laboratory Diagnosis

#### 2.3.1. Antigen Preparation from Leishmania Infantum Cultures

The crude antigen was prepared from promastigotes of *L. infantum* (IPT-1Roma) cultured in Earles modified salts medium 199 with L-glutamine and sodium bicarbonate (Euroclone, Pavia, Italy) and supplemented with 20% fetal calf serum (Euroclone, Pavia, Italy), 100 U/mL penicillin and 100 g/mL streptomycin (Sigma-Aldrich, St. Louis, MO, USA), 40 mM Hepes (Sigma-Aldrich, St. Louis, MO, USA), 0,1 mM adenine (Sigma-Aldrich, St. Louis, MO, USA) in 50 mM Hepes, 5 g/mL hemin (Sigma-Aldrich, St. Louis, MO, USA) in 50% triethanolamine, and 1 mg/mL 6-biotin (Sigma-Aldrich, St. Louis, MO, USA) in 95% ethanol. The late-log phase cultured promastigotes were harvested by centrifugation (3256× *g*, 10 min, room temperature), washed five times in phosphate-buffered saline, and lysed by repeated freezing and thawing cycles. Antigen concentration was determined using the modified Lowry method [33].

#### 2.3.2. SDS-Polyacrylamide Gel Electrophoresis and Western Blot

Serum samples were screened by WB using *L. infantum* promastigotes as antigens according to the method described elsewhere [34,35] with slight modifications. Electrophoresis was carried out using the Mini PROTEAN III Electrophoresis System (BioRad Laboratories, Hercules, CA, USA) with 14% gels under denaturing and reducing conditions with 1 mg/mL protein extracting/gel. Fractionated proteins were electroblotted at 350 mA for 1 h onto nitrocellulose sheets (BioRad Laboratories, Hercules, CA, USA). Membranes were blocked with 3% bovine serum albumin (Sigma-Aldrich, St. Louis, MO, USA) in Tris-buffered saline (TBS) (w/v) for 1 h at a temperature of 37 °C and then incubated for 3 h in a Mini PROTEAN II Multiscreen Apparatus (BioRad Laboratories, Hercules, CA, USA) with dogs’ sera diluted in a ratio of 1:10 in TBS.

After washing three times with 0.05% Tween 20 (Sigma-Aldrich, St. Louis, MO, USA) in TBS (*v*/*v*), the secondary Rabbit antibody anti-dog IgG-HRP diluted 1:6000 (Sigma-Aldrich, St. Louis, MO, USA) or Goat anti-dog IgG1-HRP diluted 1:3000 (Bethyl Laboratories, Montgomery, TX, USA) or Sheep anti-dog IgG2-HRP diluted in a ratio of 1:7500 (Bethyl Laboratories, Montgomery, TX, USA) were incubated for 1 h. Nitrocellulose membranes were washed twice, and specific anti-*Leishmania* antibodies were revealed using Amersham ECL WB detection reagents (GE Healthcare, Chalfont, UK). The resulting bands were compared using as marker Prestained Protein Molecular Weight Marker (Fermentas International Inc., ON, Canada) and Biotinylated SDS PAGE Standards broad range (BioRad Laboratories, Hercules, CA, USA). A subset of 18 samples for which serum was available before and after treatment were tested in parallel in WB to exclude possible influence of N-methyl meglumine antimoniate on polypeptide fractions response to crude antigen.

### 2.4. Statistical Analysis

The Yates test was performed on all data using EpiInfo version 6.

## 3. Results

Out of the 70 examined dogs, 25 (35.7%) responded positively to the treatment, reaching complete remission of the clinical signs and the normalization of hematological parameters. For the remaining 45 animals (64.3%), the standard treatment with N-methyl meglumine antimoniate was never allowed to reach complete disease remission. Total IgG, IgG1, and IgG2 in all 70 analyzed sera recognized a total of 27 polypeptide fractions of the *L. infantum* crude antigen, with molecular weights ranging from 10 to 286 kDa. As shown in Table 1, a significant correlation emerged between eight *L. infantum* antigens (14, 31, 33, 49, 64, 66, 99, and 169 KDa) and the total IgG in dogs with favorable prognoses. On the other hand, no significant IgG1 or IgG2 patterns could be identified. On the basis of this first set of data, the Yates corrected chi-square test has been repeated considering all eight immunodominant bands together. The results are reported in Table 2. Moreover, the different combinations between the immunodominant bands have been analyzed. We chose four antigens fractions, 31, 33, 49, and 66 kDa, as the most frequently recurrent among the examined dogs. (Table 3).

Although the non-recovered group of dogs had a statistically significant difference in the immune reaction, four antigen polypeptides of *L. infantum* were identified with molecular weights of 31, 49, 66, and 115 kDa with relative risk values of 3.6, 3.6, 2.6, and 2.5 and odds ratios of 22.67, 6.91, 7.79, and 10.50, respectively (*p* < 0.001). Out of these four antigen strains, and after 30 months’ duration of following up all the dogs, the significant difference persisted only for the 31 and 115 kDa bands with relative risk values of 1.8 and 2.65 (*p* < 0.001), respectively. Moreover, no significant IgG1 or IgG2 patterns could be identified in this group.

## 4. Discussion

The development of novel prognostic patterns for infectious diseases is an important step toward the control of this category of diseases [36,37,38]. The present study reports for the first time eight *L. infantum* antigen polypeptides (14, 31, 33, 49, 64, 66, 99, and 169 KDa) of valuable prognostic value in dogs previously infected by the parasite and treated with N-methyl meglumine antimoniate. Interestingly, the statistical data elaboration confirmed a strong association between these immunodominant bands and successful recovery in treated dogs. Among the detected antigens, fragments of 31, 49, and 66 kDa were found most frequently. More specifically, a recombinant antigen made by 31–33 kDa would be technically easier to make and still highly associated with a favorable clinical outcome.

Despite the existence of several studies on leishmaniasis, no effective vaccine is currently available for humans or dogs; thus, the control of the disease mainly relies on chemotherapeutic trials that are challenged by toxicity and/or the development of drug resistance [19,39,40]. There is a genuine need for a specific diagnostic test to distinguish between the relapsed dogs and the recovered dogs [38,41]. In fact, an understanding of the immunological mechanisms and the host immune response is very important for studying the design of novel drugs, drug targets, and vaccine preparation [17,31,42,43]. The immunological response to the *Leishmania* infection seems complex, and differs with the species that caused the infection, the virulence factors of the species, and the differences in the mechanisms of susceptibility and resistance to the infection [17,31,44,45]. Interestingly, the protective immune response to *Leishmania* infection is primarily cell-mediated, which means that it mainly involves the induction of protective immune responses and the precise migration and localization of the effector cells [16,24,46,47]. The humoral immune response has no central role in parasite clearance [31,48]; however, it has a great role in serological diagnosis, since it is expressed by high titers of IgG and IgM levels, which are highly variable before and after treatment [49,50,51,52,53,54]. Despite the existence of several studies on the relationship between cytokines, T-cell subsets, and immunoglobulin classes and subclasses, and the persistence and multiplication of *Leishmania* in experimental models and in humans [16,27,28,31,47,55], the exact mechanisms of the control immune response during and after the chemotherapy of the infected animals remain unclear, especially in dogs. Indeed, the mammalian host protection against VL is dependent on the development of Th1-type immunity, which triggers the enhanced leishmanicidal activity by the infected macrophages, whereas a Th2 response is associated with the susceptibility to infection and a strong ineffective humoral response [31,56,57,58].

As previously mentioned, both IgG1 and IgG2 are the two main immunoglobulins that have been related to host Th1/Th2 responses and disease evolution [15,23,25,26,27,28,29]. Concerning the potential prognostic value of the IgG isotypes, several studies have demonstrated that IgG1 has been associated with symptomatic infection, whereas IgG2 is more frequently found in asymptomatic dogs infected with *Leishmania* [23,26,27,29,30,32,54,59]. In this regard, it was reported that immune reactions in the early phases of infection (IgG2) recognize mainly low-weight polypeptides, such as 14, 16, and 18 kDa antigen strains, while the immune reactions against 24 kDa polypeptides characterize the early symptomatic stages of the disease [16,23]. In symptomatic dogs, IgG2 reacts against seven main antigen strains: 26, 29, 34–35, 42, 45, 50–57, 67 kDa; however, the 67 kDa fragment is also the main target for IgG1 [27,60]. The levels of both immunoglobulin and IgG2 are drastically decreased after treatment, especially IgG1 and IgG2 anti-67 kDa [60]. Given the above information, the use of an accurate serological technique to check the alteration in IgG and IgG2 levels during the course of the infection and/or after treatment might be of promising prognostic value [16,30,59]. Several published works have revealed the major role played by many serological tests in the diagnosis of the disease [4,61,62]. Among the other serological assays, WB has been identified as a highly sensitive and specific serological test for the detection of specific anti-*Leishmania* antibodies in many animals, especially in dogs [34,35,63,64,65,66]. However, some previous studies on symptomatic dogs have distinguished between the immunodominant and the non-immunodominant protein bands through the WB analysis of antibody response during experimental canine *L. infantum* infection [60,67]. In the same study of 12 antigenic bands examined, including the immunodominant bands (12, 14, 24, 29, 48, and 68 kDa), it revealed significantly increased intensities and higher frequencies of recognition compared to the non-immunodominant bands [67]. Reactivity with the 14, 48, and 68 kDa bands signified early infection, whereas increased reactivity with the 14, 24, and 29 kDa bands was associated with post-treatment parasite persistence and potential unfavorable prognosis, which was consistent with our results [67].

As depicted in Table 1, Table 2 and Table 3, several antigenic molecules have been detected using WB, which is consistent with the findings of some studies on sera from dogs challenged with wild-type *L. infantum* with prior vaccination with *L. infantum* H-line, which concluded on the valuable diagnostic role of various antigenic molecules, i.e., 21, 85, and 110 kDa of *L. infantum* in dogs infected with VL [63,66,68,69,70,71]. Another study concluded on the high sensitivity (99.1%) of WB in the detection of several bands between 15 and 118 kDa in two groups of CL patients, especially the 63 kDa band, which was found to be more sensitive [66,68,69]. Based on these findings, we also suggest that the control of infection after successful chemotherapy in dogs involves a particular and specific antibody response and clear statistical correlation with the detected bands and total IgG level (not IgG1 or IgG2), which is consistent with some previous studies that concluded that a measurable antibody response was detected in most cases of CL and VL [24,28,66,72].

## 5. Conclusions

Given the above data, these *L. infantum* antigenic fragments weighting can be considered as favorable prognostic indicators and might play an important role in the modulation of the host’s immune response. These findings also suggest that WB analysis of antibodies against those detected antigens represents a specific prognostic test for distinguishing between dogs that recovered from the infection and those that did not recover. In addition to its diagnostic–prognostic value, a valuable future application of our data is a recombinant antigen made out of the eight indicated fragments, which could be very promising in the implementation of novel canine vaccines that can be used later in humans for combating this neglected disease. Further research is suggested to further explore the topic on a large-scale level in other reservoirs and various parasite species.

## Figures and Tables

**Table 1:** Odds ratio values by the Yates chi-square test for eight significant antigen strains of *L. infantum*.

Ag Weight (kDa)	Odds Ratio (95%CI)	χ^2^	*p*-Values
169	5.74 (1.70–20.25)	8.96	0.0027595
99	8.00 (2.23–30.48)	12.2	0.000479
66	5.49 (1.45–22.58)	6.95	0.0084019
64	9.79 (2.76–36.65)	15.4	0.0000869
49	4.33 (1.30–15.05)	6.06	0.0135914
33	4.75 (1.42–16.57)	6.98	0.008259
31	5.02 (1.32–20.65)	6.1	0.0134882
14	13.81 (3.63–56.39)	19.36	0.0000108

Ag: antigen; CI: confidence interval and χ^2^: Yates corrected chi-square.

**Table 2:** Odds ratio values by the Yates chi-square test for all eight significant antigen strains of *L. infantum* considered together.

Band weights: 14, 31, 33, 49, 64, 66, 99, and 169 KDa			
	Bands Present	Bands Absent	
Recovered	12	13	25
Not recovered	1	44	45
	13	57	70
Odds ratio (95% CI) = 40.62; OR interval (4.62–917.57)			
χ^2^ *p*			
Yates corrected: 19.35 Fisher 2-tailed 0.0000108			

OR: odds ratio; CI: confidence interval; χ^2^: Yates corrected chi-square.

**Table 3:** Association levels expressed by OR (CI 95%) and Yates chi-square test between different combinations of immunodominant antigens.

Ag Weight (kDa)	Odds Ratio(95%CI)	χ ^2^	*p*-Values
31–33	8.71 (2.49–32.07)	13.92	0.0001911
31–49	7.79 (2.25–28.31)	12.54	0.0003988
31–66	6.00 (1.70–22.44)	8.81	0.0029914
33–49	7.07 (2.10–24.83)	11.70	0.0006253
33–66	7.95 (2.33–28.38)	13.08	0.0002980
49–66	8.71 (2.49–32.07)	13.92	0.0001911
31–33–49	9.00 (2.59–32.81)	14.58	0.0001341
31–33–66	9.00 (2.59–32.81)	14.58	0.0001341
31–49–66	8.71 (2.49–32.07)	13.93	0.0001911
33–49–66	9.00 (2.59–32.81)	14.58	0.0001341
31–33–49–66	9.00 (2.59–32.81)	14.58	0.0001341

Ag: antigen; CI: confidence interval and χ^2^: Yates corrected chi-square.

## Data Availability

Blood samples from dogs were collected by licenced veterinarians within routine clinical diagnostic activities and after informed consent of the animals’ owner.

## References

[1] Clem A. (2010). A current perspective on leishmaniasis. J. Glob. Infect. Dis..

[2] Desjeux P., Alvar J. (2003). Leishmania/HIV co-infections: Epidemiology in Europe. Ann. Trop. Med. Parasitol..

[3] World Health Organization (WHO) (2014). Leishmaniasis Fact Sheet N°375″.

[4] Elmahallawy E.K., Sampedro Martinez A., Rodriguez-Granger J., Hoyos-Mallecot Y., Agil A., Navarro Mari J.M., Gutierrez Fernandez J. (2014). Diagnosis of leishmaniasis. J. Infect. Dev. Ctries..

[5] Monge-Maillo B., Norman F.F., Cruz I., Alvar J., Lopez-Velez R. (2014). Visceral leishmaniasis and HIV coinfection in the Mediterranean region. PLoS Negl. Trop. Dis..

[6] Maroli M., Rossi L., Baldelli R., Capelli G., Ferroglio E., Genchi C., Gramiccia M., Mortarino M., Pietrobelli M., Gradoni L. (2008). The northward spread of leishmaniasis in Italy: Evidence from retrospective and ongoing studies on the canine reservoir and phlebotomine vectors. Trop. Med. Int. Health.

[7] Elmahallawy E.K., Cuadros-Moronta E., Liebana-Martos M.C., Rodriguez-Granger J.M., Sampedro-Martinez A., Agil A., Navarro-Mari J.M., Bravo-Soto J., Gutierrez-Fernandez J. (2015). Seroprevalence of Leishmania infection among asymptomatic renal transplant recipients from southern Spain. Transpl. Infect. Dis..

[8] Biglino A., Bolla C., Concialdi E., Trisciuoglio A., Romano A., Ferroglio E. (2010). Asymptomatic Leishmania infantum infection in an area of northwestern Italy (Piedmont region) where such infections are traditionally nonendemic. J. Clin. Microbiol..

[9] Postigo J.A. (2010). Leishmaniasis in the World Health Organization Eastern Mediterranean Region. Int. J. Antimicrob. Agents.

[10] Morosetti G., Bongiorno G., Beran B., Scalone A., Moser J., Gramiccia M., Gradoni L., Maroli M. (2009). Risk assessment for canine leishmaniasis spreading in the north of Italy. Geospat. Health.

[11] Elmahallawy E.K., Zanet S., Poggi M., Alsharif K.F., Agil A., Trisciuoglio A., Ferroglio E. (2021). Feline Leishmaniosis in Northwestern Italy: Current status and zoonotic implications. Vet. Sci..

[12] Ferroglio E., Maroli M., Gastaldo S., Mignone W., Rossi L. (2005). Canine leishmaniasis, Italy. Emerg. Infect. Dis..

[13] Galvez R., Montoya A., Cruz I., Fernandez C., Martin O., Checa R., Chicharro C., Miguelanez S., Marino V., Miro G. (2020). Latest trends in Leishmania infantum infection in dogs in Spain, Part I: Mapped seroprevalence and sand fly distributions. Parasit. Vectors.

[14] Solano-Gallego L., Koutinas A., Miro G., Cardoso L., Pennisi M.G., Ferrer L., Bourdeau P., Oliva G., Baneth G. (2009). Directions for the diagnosis, clinical staging, treatment and prevention of canine leishmaniosis. Vet. Parasitol..

[15] Gradoni L. (2015). Canine Leishmania vaccines: Still a long way to go. Vet. Parasitol..

[16] Barbieri C.L. (2006). Immunology of canine leishmaniasis. Parasite Immunol..

[17] Elmahallawy E.K., Alkhaldi A.A.M. (2021). Insights into Leishmania molecules and their potential contribution to the virulence of the parasite. Vet. Sci..

[18] Moreno J., Alvar J. (2002). Canine leishmaniasis: Epidemiological risk and the experimental model. Trends Parasitol..

[19] Elmahallawy E.K., Agil A. (2015). Treatment of leishmaniasis: A review and assessment of recent research. Curr. Pharm. Des..

[20] Reguera R.M., Elmahallawy E.K., Garcia-Estrada C., Carbajo-Andres R., Balana-Fouce R. (2019). DNA topoisomerases of Leishmania parasites; druggable targets for drug discovery. Curr. Med. Chem..

[21] Elmahallawy E.K., Jimenez-Aranda A., Martinez A.S., Rodriguez-Granger J., Navarro-Alarcon M., Gutierrez-Fernandez J., Agil A. (2014). Activity of melatonin against Leishmania infantum promastigotes by mitochondrial dependent pathway. Chem. Biol. Interact..

[22] Zheoat A.M., Alenezi S., Elmahallawy E.K., Ungogo M.A., Alghamdi A.H., Watson D.G., Igoli J.O., Gray A.I., de Koning H.P., Ferro V.A. (2021). Antitrypanosomal and antileishmanial activity of chalcones and flavanones from *Polygonum salicifolium*. Pathogens.

[23] Iniesta L., Gallego M., Portus M. (2007). Idiotype expression of IgG1 and IgG2 in dogs naturally infected with *Leishmania infantum*. Vet. Immunol. Immunopathol..

[24] Daneshvar H., Mahmmodi Z., Kamiabi H., Phillips R.S., Burchmore R. (2014). Dogs vaccinated with gentamicin-attenuated Leishmania infantum or infected with wild-type parasite can be distinguished by Western blotting. Parasite Immunol..

[25] Leandro C., Santos-Gomes G.M., Campino L., Romao P., Cortes S., Rolao N., Gomes-Pereira S., Rica Capela M.J., Abranches P. (2001). Cell mediated immunity and specific IgG1 and IgG2 antibody response in natural and experimental canine leishmaniosis. Vet. Immunol. Immunopathol..

[26] Nieto C.G., Garcia-Alonso M., Requena J.M., Miron C., Soto M., Alonso C., Navarrete I. (1999). Analysis of the humoral immune response against total and recombinant antigens of Leishmania infantum: Correlation with disease progression in canine experimental leishmaniasis. Vet. Immunol. Immunopathol..

[27] Quinnell R.J., Courtenay O., Garcez L.M., Kaye P.M., Shaw M.A., Dye C., Day M.J. (2003). IgG subclass responses in a longitudinal study of canine visceral leishmaniasis. Vet. Immunol. Immunopathol..

[28] Solano-Gallego L., Riera C., Roura X., Iniesta L., Gallego M., Valladares J.E., Fisa R., Castillejo S., Alberola J., Ferrer L. (2001). *Leishmania infantum*-specific IgG, IgG1 and IgG2 antibody responses in healthy and ill dogs from endemic areas. Evolution in the course of infection and after treatment. Vet. Parasitol..

[29] Deplazes P., Smith N.C., Arnold P., Lutz H., Eckert J. (1995). Specific IgG1 and IgG2 antibody responses of dogs to *Leishmania infantum* and other parasites. Parasite Immunol..

[30] Oliveira T.M., Mineo T.W., Bason M., Day M.J., Machado R.Z. (2009). IgG subclass profile of serum antibodies to Leishmania chagasi in naturally infected and vaccinated dogs. Vet. Parasitol..

[31] Elmahallawy E.K., Alkhaldi A.A.M., Saleh A.A. (2021). Host immune response against leishmaniasis and parasite persistence strategies: A review and assessment of recent research. Biomed. Pharmacother..

[32] Iniesta L., Gallego M., Portus M. (2005). Immunoglobulin G and E responses in various stages of canine leishmaniosis. Vet. Immunol. Immunopathol..

[33] Lowry O.H., Rosebrough N.J., Farr A.L., Randall R.J. (1951). Protein measurement with the Folin phenol reagent. J. Biol. Chem..

[34] Ferroglio E., Centaro E., Mignone W., Trisciuoglio A. (2007). Evaluation of an ELISA rapid device for the serological diagnosis of *Leishmania infantum* infection in dog as compared with immunofluorescence assay and Western blot. Vet. Parasitol..

[35] Ferroglio E., Trisciuoglio A., Gastaldo S., Mignone W., Trisciuoglio A. (2002). Comparison of ELISA, IFAT and Western blot for the serological diagnosis of Leishmania infantum infection in dog. Parassitologia.

[36] Van Seventer J.M., Hochberg N.S. (2017). Principles of infectious diseases: Transmission, diagnosis, prevention, and control. Int. Encycl. Public Health.

[37] Pereira M.A., Santos R., Oliveira R., Costa L., Prata A., Goncalves V., Roquette M., Vala H., Santos-Gomes G. (2020). Prognostic factors and life expectancy in canine Leishmaniosis. Vet. Sci..

[38] Duthie M.S., Lison A., Courtenay O. (2018). Advances toward diagnostic tools for managing zoonotic visceral Leishmaniasis. Trends Parasitol..

[39] Sereno D., Maia C., Ait-Oudhia K. (2012). Antimony resistance and environment: Elusive links to explore during *Leishmania* life cycle. Int. J. Parasitol. Drugs Drug Resist..

[40] Sundar S. (2001). Drug resistance in Indian visceral leishmaniasis. Trop. Med. Int. Health.

[41] Solano-Gallego L., Cardoso L., Pennisi M.G., Petersen C., Bourdeau P., Oliva G., Miró G., Ferrer L., Baneth G. (2017). Diagnostic challenges in the era of canine *Leishmania infantum* vaccines. Trends Parasitol..

[42] Okwor I., Uzonna J. (2008). Persistent parasites and immunologic memory in cutaneous leishmaniasis: Implications for vaccine designs and vaccination strategies. Immunol. Res..

[43] Vanloubbeeck Y., Jones D.E. (2004). The immunology of *Leishmania* infection and the implications for vaccine development. Ann. N.Y. Acad. Sci..

[44] Ehrlich A., Castilho T.M., Goldsmith-Pestana K., Chae W.J., Bothwell A.L., Sparwasser T., McMahon-Pratt D. (2014). The immunotherapeutic role of regulatory T cells in *Leishmania (Viannia)* panamensis infection. J. Immunol..

[45] McMahon-Pratt D., Alexander J. (2004). Does the Leishmania major paradigm of pathogenesis and protection hold for New World cutaneous leishmaniases or the visceral disease?. Immunol. Rev..

[46] Selvapandiyan A., Dey R., Gannavaram S., Lakhal-Naouar I., Duncan R., Salotra P., Nakhasi H.L. (2012). Immunity to visceral leishmaniasis using genetically defined live-attenuated parasites. J. Trop. Med..

[47] Reis A.B., Martins-Filho O.A., Teixeira-Carvalho A., Giunchetti R.C., Carneiro C.M., Mayrink W., Tafuri W.L., Correa-Oliveira R. (2009). Systemic and compartmentalized immune response in canine visceral leishmaniasis. Vet. Immunol. Immunopathol..

[48] Vannier-Santos M.A., Martiny A., de Souza W. (2002). Cell biology of Leishmania spp.: Invading and evading. Curr. Pharm. Des..

[49] Liew F.Y., O’Donnell C.A. (1993). Immunology of leishmaniasis. Adv. Parasitol..

[50] Galvao-Castro B., Sa Ferreira J.A., Marzochi K.F., Marzochi M.C., Coutinho S.G., Lambert P.H. (1984). Polyclonal B cell activation, circulating immune complexes and autoimmunity in human american visceral leishmaniasis. Clin. Exp. Immunol..

[51] Rolland-Burger L., Rolland X., Grieve C.W., Monjour L. (1991). Immunoblot analysis of the humoral immune response to Leishmania donovani infantum polypeptides in human visceral leishmaniasis. J. Clin. Microbiol..

[52] Lambertz U., Silverman J.M., Nandan D., McMaster W.R., Clos J., Foster L.J., Reiner N.E. (2012). Secreted virulence factors and immune evasion in visceral leishmaniasis. J. Leukoc. Biol..

[53] Dos Santos J.I., Morgado M.G., Galvao-Castro B. (1987). Human visceral leishmaniasis: Analysis of the specificity of humoral immune response to polypeptides of Leishmania donovani chagasi. Am. J. Trop. Med. Hyg..

[54] Rodriguez A., Solano-Gallego L., Ojeda A., Quintana J., Riera C., Gallego M., Portus M., Alberola J. (2006). Dynamics of Leishmania-specific immunoglobulin isotypes in dogs with clinical leishmaniasis before and after treatment. J. Vet. Intern. Med..

[55] Strauss-Ayali D., Baneth G., Shor S., Okano F., Jaffe C.L. (2005). Interleukin-12 augments a Th1-type immune response manifested as lymphocyte proliferation and interferon gamma production in Leishmania infantum-infected dogs. Int. J. Parasitol..

[56] Kaushal H., Bras-Goncalves R., Negi N.S., Lemesre J.L., Papierok G., Salotra P. (2014). Role of CD8^+^ T cells in protection against Leishmania donovani infection in healed Visceral Leishmaniasis individuals. BMC Infect. Dis..

[57] Sharma U., Singh S. (2009). Immunobiology of leishmaniasis. Indian J. Exp. Biol..

[58] Khadem F., Uzonna J.E. (2014). Immunity to visceral Leishmaniasis: Implications for immunotherapy. Future Microbiol..

[59] Marcondes M., Ikeda F.A., Vieira R.F., Day M.J., Lima V.M., Rossi C.N., Perri S.H., Biondo A.W. (2011). Temporal IgG subclasses response in dogs following vaccination against *Leishmania* with Leishmune(R). Vet. Parasitol..

[60] Fernandez-Perez F.J., Gomez-Munoz M.T., Mendez S., Alunda J.M. (2003). *Leishmania*-specific lymphoproliferative responses and IgG1/IgG2 immunodetection patterns by Western blot in asymptomatic, symptomatic and treated dogs. Acta Trop..

[61] Antinori S., Schifanella L., Corbellino M. (2012). Leishmaniasis: New insights from an old and neglected disease. Eur. J. Clin. Microbiol. Infect. Dis..

[62] Chappuis F., Sundar S., Hailu A., Ghalib H., Rijal S., Peeling R.W., Alvar J., Boelaert M. (2007). Visceral Leishmaniasis: What are the needs for diagnosis, treatment and control?. Nat. Rev. Microbiol..

[63] Daneshvar H., Namazi M.J., Kamiabi H., Burchmore R., Cleaveland S., Phillips S. (2014). Gentamicin-attenuated *Leishmania infantum* vaccine: Protection of dogs against canine visceral leishmaniosis in endemic area of southeast of Iran. PLoS Negl. Trop. Dis..

[64] Aisa M.J., Castillejo S., Gallego M., Fisa R., Riera M.C., de Colmenares M., Torras S., Roura X., Sentis J., Portus M. (1998). Diagnostic potential of Western blot analysis of sera from dogs with leishmaniasis in endemic areas and significance of the pattern. Am. J. Trop. Med. Hyg..

[65] Neogy A.B., Vouldoukis I., Silva O.A., Tselentis Y., Lascombe J.C., Segalen T., Rzepka D., Monjour L. (1992). Serodiagnosis and screening of canine visceral leishmaniasis in an endemic area of Corsica: Applicability of a direct agglutination test and immunoblot analysis. Am. J. Trop. Med. Hyg..

[66] Zeyrek F.Y., Korkmaz M., Ozbel Y. (2007). Serodiagnosis of Anthroponotic Cutaneous Leishmaniasis (ACL) caused by *Leishmania tropica* in Sanliurfa Province, Turkey, where ACL Is highly endemic. Clin. Vaccine Immunol..

[67] Talmi-Frank D., Strauss-Ayali D., Jaffe C.L., Baneth G. (2006). Kinetics and diagnostic and prognostic potential of quantitative Western blot analysis and antigen-specific enzyme-linked immunosorbent assay in experimental canine leishmaniasis. Clin. Vaccine Immunol..

[68] Marty P., Lelievre A., Quaranta J.F., Suffia I., Eulalio M., Gari-Toussaint M., Le Fichoux Y., Kubar J. (1995). Detection by Western blot of four antigens characterizing acute clinical leishmaniasis due to *Leishmania infantum*. Trans. R Soc. Trop. Med. Hyg..

[69] Mary C., Ange G., Dunan S., Lamouroux D., Quilici M. (1993). Characterization of a circulating antigen involved in immune complexes in visceral leishmaniasis patients. Am. J. Trop. Med. Hyg..

[70] Olías-Molero A.I., Corral M.J., Jiménez-Antón M.D., Alunda J.M. (2019). Early antibody response and clinical outcome in experimental canine leishmaniasis. Sci. Rep..

[71] Lasri S., Sahibi H., Natami A., Rhalem A. (2003). Western blot analysis of *Leishmania infantum* antigens using sera from pentamidine-treated dogs. Vet. Immunol. Immunopathol..

[72] Teixeira Neto R.G., Giunchetti R.C., Carneiro C.M., Vitor R.W., Coura-Vital W., Quaresma P.F., Ker H.G., de Melo L.A., Gontijo C.M., Reis A.B. (2010). Relationship of Leishmania-specific IgG levels and IgG avidity with parasite density and clinical signs in canine leishmaniasis. Vet. Parasitol..

